# Light on the sustainable preparation of aryl-cored dibromides

**DOI:** 10.3762/bjoc.20.95

**Published:** 2024-05-14

**Authors:** Fabrizio Roncaglia, Alberto Ughetti, Nicola Porcelli, Biagio Anderlini, Andrea Severini, Luca Rigamonti

**Affiliations:** 1 University of Modena and Reggio Emilia, Department of Chemical and Geological Sciences, Via Campi 103, 41125 Modena, Italy

**Keywords:** aryl halides, benzyl halides, bromination, sustainability

## Abstract

Both aryl and benzyl polybromides have gained significant importance as reactive building blocks in polymer and materials chemistry. Their preparation primarily relies on established synthetic methods using molecular bromine or *N*-bromosuccinimide, known for their reliability and effectiveness. However, from a sustainability perspective, these methods suffer from the generation of stoichiometric amounts of byproducts and often encounter selectivity troubles. To mitigate these issues, we extended the greener peroxide-bromide halogenation method, initially developed for monobromides, to afford aryl-cored polybromides in high yields. The same method can be employed in two variants modulated by light irradiation. This external switch can be used to selectively trigger side-chain or core halogenation.

## Introduction

Activation through halogens has become a key strategy in achieving desired reactivity. Substitution of a hydrogen atom with a halogen atom within an organic skeleton significantly increases the electrophilicity of the linked carbon centre, enhancing concerted (S_N_2) as well as carbenium ion-mediated (S_N_1) substitutions, common – for instance – on benzylic positions. Useful alkenyl functional groups can also be obtained by means of elimination processes, if proper alkyl side chains are present. Additional opportunities offered by C(sp^3^)–Hal bonds arise from the ease of their homolytic cleavage, leading to the formation of reactive carbon-centred radicals. C(sp^2^)–Hal bonds of aryl halides also exhibit high reactivity, particularly towards transition-metal-mediated cross-coupling processes or Ar-S_N_ reactions.

Benzyl and aryl halides, collectively referred to as 'aryl-cored halides', have found extensive applications across various fields, including synthesis [1], photonics [2], and diagnostics [3]. Their popularity stems from their structural rigidity, potential conjugation with the remaining structure, and the capability to form additional π–π-stacking interactions. Dihalides, in particular, are highly preferred in such applications, enabling the formation of molecular wires or extended structures – either linear or cyclic – with enhanced functionality. Consequently, they play a crucial role in the fabrication of polymers [4–7], cyclophanes [8], photoactive materials [9–11], membranes [12], and other architectures.

The synthetic potential of aryl-cored halides can be broadened by converting C–Hal functions into different functional groups. For example, aldehyde and amine functionalities can be readily derived from C(sp^3^)–Hal functions through hydrolysis–oxidation [13] or substitution [14], respectively. This is of significant interest in the context of covalent organic frameworks (COFs) and metal-organic frameworks (MOFs), frequently assembled through imine linkages.

While C–H activation through halogens presents clear technical advantages, it also brings forth concerns about the toxicity of halo compounds to both human health and the environment [15]. Nevertheless, in line with the European Union's 'green new deal' guidelines [16], addressing two pivotal issues could facilitate the environmentally conscious utilisation of halogenated compounds as intermediates in chemical processes:

1. The development of more sustainable production methods for halo compounds, potentially involving the use of eco-friendly halogenation reagents.
2. The development of methods for the efficient removal and recycle of halogens, advocating principles of a circular economy.

It is noteworthy that both aspects are influenced by the type of halogen employed. When considering the most atom-economical options, namely chlorine and bromine, the latter typically exhibits some advantages over the former. These include: (i) better regioselectivity in radical processes, attributed to the lower bond enthalpy of H–Br (88 kcal/mol) compared to H–Cl (103 kcal/mol) [17], (ii) greater electrophilicity of the halo compound due to better leaving group ability of the halide ion, (iii) reduced toxicity, presumably due to faster hydrolysis [18], and (iv) easier oxidation of the halide to molecular halogen (*E*^0^ = 1.087 V (SHE) for Br_2_/Br^−^; *E*^0^ = 1.358 V (SHE) for Cl_2_/Cl^−^) [19] resulting in easier recyclability.

**Figure 1:**
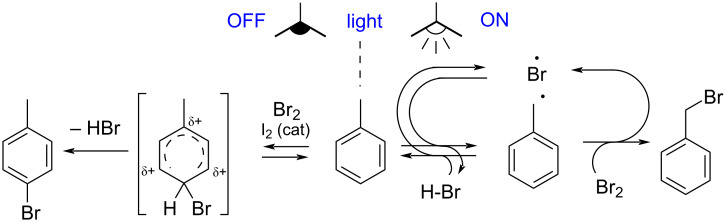
Comparison between the light-initiated radical halogenation of toluene (right), and the Ar-S_E_ bromination in the dark (left).

Light irradiation often significantly influences the selectivity of halogenation processes. Photolytic cleavage of molecular halogens gives rise to radicals that are known to favour benzylic functionalisation [17]. Conversely, the same molecular halogens exhibit prominent functionalisation on the aromatic ring when used in the dark [20]. A classic example is the bromination of toluene with molecular bromine. When the system is exposed to light (right side of Figure 1), a radical mechanism is initiated by Br^•^ coming from Br_2_ homolysis. Propagation involves the reversible abstraction of a benzylic hydrogen atom from the substrate by Br^•^, to give HBr and a structure-stabilised carbon-centred radical, which may react with Br_2_ to give the brominated product, thus regenerating Br^•^ that is able to sustain the chain process. In the absence of light (left side of Figure 1), the reaction follows a different mechanism, producing the *ortho* and *para*-bromoarenes through Ar-S_E_, that involves cationic intermediates. In this case, a catalytic amount of iodine [21–22] or FeCl_3_ [23] is added to enhance the electrophilicity of bromine.

While widely employed and capable of producing reliable results on various substrates, the direct use of molecular bromine poses sustainability challenges. Its innate reactivity requires stringent safety protocols during transportation, storage, and handling [24]. A related concern involves the stability of the diluting media, often requiring the use of toxic CCl_4_ to prevent undesired solvent degradation. Furthermore, the direct use of Br_2_, even if used in low concentration, exhibits limited selectivity towards benzylic bromination, primarily due to co-bromination occurring on the aromatic ring. This side process produces awkward halogenated byproducts that can complicate product separation, and will require disposal. Lastly, only one half of the halogen load is incorporated into the product, the other half being lost as bromide ion.

**Figure 2:**
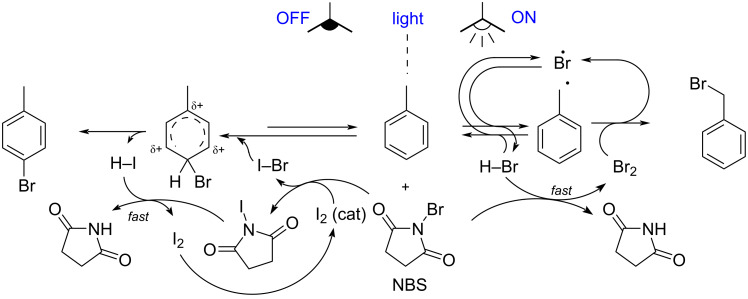
Toluene halogenation mediated by NBS in absence (left) or exposed to light (right).

A solution for some of the aforementioned problems is the in situ generation of bromine: firstly, the handling of Br_2_ is no longer an issue, since it is formed inside the reaction vessel, secondly this approach allows enhanced selectivity of the bromination, as the amount and timing of the chemical generation can be modulated. In addition, in situ regeneration of bromine from bromide byproducts improves the atom economy of the overall process. A pertinent example of this technique is represented by the Wohl–Ziegler halogenation protocol, which is based on the stable and easily handled *N*-bromosuccinimide (NBS), which is able to slowly deliver molecular bromine through its interaction with bromide ions [17,25]. This method found prevalent application in the bromination of side-chain positions (right side of Figure 2) [26–27]. However, the addition of molecular iodine in catalytic amounts makes it suitable for aromatic bromination “in the dark” (left side of Figure 2). This gives rise to a radical-initiated Ar-S_E_ mechanism, which is reported to proceed through the generation of a mixed molecular halogen [28]. As an alternative to iodine, the trityl cation [29] is reported too. Additional benefits of the method include its ability to work in neutral conditions, and the potential quantitative incorporation of the halogen into the product (no bromide byproducts are generated).

Unfortunately, the safety and selectivity advantages of using NBS are counterbalanced by other issues. Specifically, light irradiation is often ineffective as an initiator for NBS [26,30], introducing the necessity to add a radical initiator, such as azobisisobutyronitrile (AIBN) [31–34] or benzoyl peroxide [35–36]; substances affected by safety concerns on transportation, storage, and use. Moreover, the regeneration of the halogen comes at a cost: the release of stoichiometric amounts of succinimide, whose recovery requires additional chemical operations, and reconversion to NBS is typically accomplished with stoichiometric Br_2_ [37–38]. Recently, certain bromine carriers, potentially offering improvements over NBS, have been proposed [24], demonstrating notable success in specific halogenation reactions. Nevertheless, additional studies are required to broaden the scope of most alternatives and to evaluate options for carrier recovery/regeneration.

Regardless of the employed method, the concept of (re-)generating molecular bromine from “spent” bromide ions is a crucial idea to improve the atom economy of the process. Therefore, various forms of oxidative halogenations have been reported over the years. For example, Ishii described the use of the NaBrO_3_–NaHSO_3_ couple to slowly generate Br_2_ through an aqueous redox equilibrium [39]. Few years later, Adimurthy et al. proposed an improved variant based on the redox couple NaBr–NaBrO_3_ in acidic media [40–41]. Other variations include the system KBr–Oxone^®^ [42].

However, based on a literature review, we concluded that unparalleled efficiency and sustainability can be achieved through the well-established redox equilibria between hydrogen peroxide and halide ion in aqueous acidic media (Equation 1) [43]. Its practical implementation is surprisingly simple as standard aqueous HBr and H_2_O_2_ solutions are effective. The generated bromine can be involved in different chemical mechanisms, such as an Ar-S_E_ (Equation 2) [44] or a radical substitution on activated positions (Equation 3). In the context of the radical process, it is noteworthy that a standard household LED lamp can serve as an efficient initiator, capable of triggering bromine photolysis (Equation 3, top) [45]. Hence, no oxidant-derived residues apart from water are formed and, at the end of the process, the residual halogen (if any) can decompose hydrogen peroxide into molecular oxygen (Equation 4) [45].


[1]

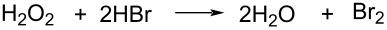




[2]

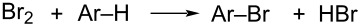




[3]

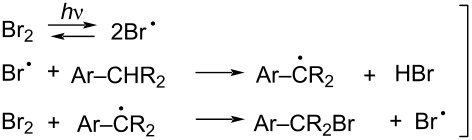




[4]

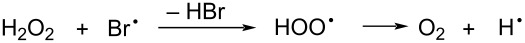



Over the years, various peroxide-bromide processes have been developed based on this chemistry. In particular, significant attention has been given to the preparation of benzyl (mono)bromides [46–49], which can be produced with high efficiency. The peroxide-bromide method appears to be especially suitable for obtaining aryl-cored dibromides, too. However, to the best of our knowledge, this option has been scarcely exploited, except for a few reports on some activated arenes [50–51].

**Figure 3:**
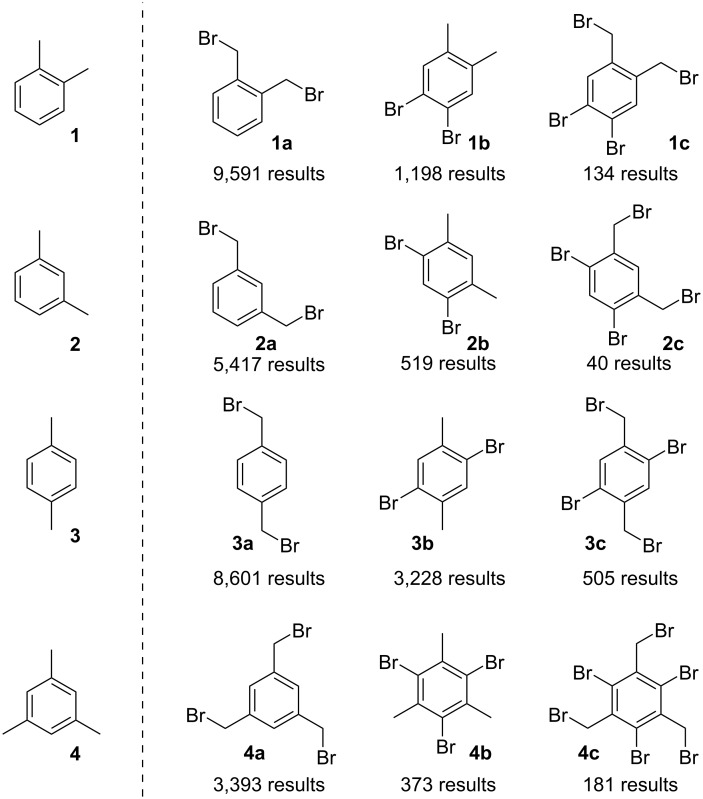
Scifinder^®^ reaction hits for the structure “as drawn” (January 2024).

Based on a SciFinder^®^ survey, we came to realise that a small number of aryl-cored dibromides, which can be derived from xylenes (**1**, **2**, **3**) or mesitylene (**4**, Figure 3), play a predominant role as building blocks for the construction of functional materials and/or polymeric architectures. Some aryl/benzyl polybromides are also well known. Looking at this subject, the number of found reaction hits (role = reagent) for the structure “as drawn” provides a quantitative measurement of the synthetic usefulness of these halides.

This work focuses on the application of the peroxide-bromide method to the synthesis of aryl-cored polybromides obtainable from xylenes or mesitylene. The selected targets are those of heightened interest to the scientific community, identified as those with a score of at least 100 reaction hits in the Scifinder^®^ search (Figure 3).

## Results and Discussion

As *p*- and *o*-xylenes represent a primary source for aryl-cored halides, our investigation started with the conversion of **3** into **3a**, taking as “zero-point” conditions those reported for monobromides [48–49], with the only change of a double amount of brominating agent (HBr·2H_2_O_2_). A household white LED lamp was used as the activator, and hydrogen peroxide was slowly added, by means of a syringe pump. As the bromine colour generated after each H_2_O_2_ drop disappeared quickly, the reaction time was reduced from 24 h to 8 h. In these conditions, the process exhibited complete conversion, but strong selectivity in favour of the monohalide **3am** (entry 1, Table 1).

**Table 1:** Light-mediated bromination of *p*-xylene with in situ-generated Br_2_.^a^

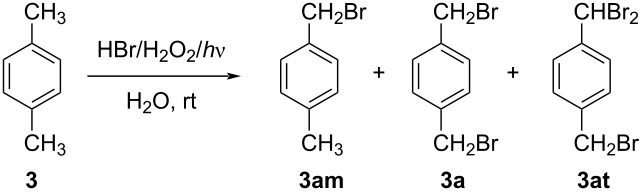							

entry	H_2_O_2_ dropping time (h)	post- dropping time (h)	H_2_O (mL)	CH_2_Cl_2_ (mL)	**3am** (%)	**3a** (%)	**3at** (%)

1	8.0	–	4.0	–	78	22	traces
2	8.0	–	1.0	–	18	26	56
3	3.0	1.0	1.0	–	63	37	1
4	3.0	1.0	2.0	–	52	46	2
5	3.0	1.0	2.0	1.0	20	74	6
6	3.0	1.0	2.0	0.5	21	74	5
7	2.0	1.5	2.0	0.5	9	86	5
8	2.0	1.5	1.5	1.0	1	90	9

^a^Reaction conditions: *p*-xylene (531 mg, 5 mmol), H_2_O (see Table), HBr (48 wt % aqueous solution, 1.25 mL, 11 mmol), white LED lamp, H_2_O_2_ (35 wt % aqueous solution, 2.00 mL, 23.3 mmol) slowly dropped by a syringe pump (see Table), CH_2_Cl_2_ (see Table). Yields are reported as molar % obtained from ^1^H NMR spectra.

The reduction of the volume of the aqueous phase (Table 1, entry 2) resulted in a small shift towards the desired product **3a** but also in the predominant overreaction towards tribromide **3at**. Formation of **3at** was substantially controlled by reducing the reaction time (4 h overall, entries 3 and 4 in Table 1). In these last experiments a whitish solid appeared during addition of the oxidant, initially observed in the one with lower water amount (Table 1, entry 3). The ^1^H NMR confirmation of the nature of the solid as **3a**, makes clear that phase separation was an important issue, resulting in a limitation of reagents’ diffusion. The addition of a small amount of CH_2_Cl_2_ was thus considered (Table 1, entries 5 and 6) and resulted in a substantial selectivity improvement. The achievement of a better homogeneity, let it possible to further reduce the reaction time (Table 1, entry 7), giving total conversion and excellent selectivity toward **3a**. Water is an essential constituent of the system, as its polar nature favours the extraction of HBr from the organic phase, resulting in a useful shift of the reversible hydrogen abstraction from the substrate (Figure 1 and Equation 3, middle) [45]. This two-phase system is likely to benefit from efficient stirring as well as from a proper volume ratio between aqueous and organic phases, implemented in entry 8, which resulted in further enhancement of selectivity toward **3a**.

Different wavelengths were also evaluated, through the use of different types of lamps, particularly those with partial UV emission. In a specific implementation, in consideration of the glass filter’s capabilities, some UV emitting diodes were inserted inside the vessel. Anyway, no significant modulation was observed, probably due to the negligible adsorption of Br_2_ at wavelengths shorter than 380 nm [52].

We finally evaluated a variation in the H_2_O_2_/HBr molar ratio to determine the optimal amount of H_2_O_2_, typically used in a twofold molar ratio relative to HBr [49]. These experiments raised on the observation of gas evolution (presumably O_2_) starting when the added moles of H_2_O_2_ reached that of HBr. In order to confirm that this phenomenon was not attributable exclusively to the undesired peroxide decomposition described in Equation 4, we repeated the same reaction on *p*-xylene as in entry 8 of Table 1, but with different H_2_O_2_/HBr molar ratios. Our data confirmed that using reduced amounts of peroxide (in relation to H_2_O_2_/HBr = 2.0) leads to proportionally reduced conversion with remaining unreacted **3** (Supporting Information File 1, Table S1, entries 1, 2 and 3). The reason can be ascribed to the partial physiological peroxide decomposition due to the presence of both bromine and bromide in acid media [43]. Besides, an increased amount of peroxide did not show any noticeable benefits in selectivity toward **3a**, but it led to increased formation of over-halogenated products (Table S1, entries 4 and 5 in Supporting Information File 1).

The developed method was finally successfully scaled up to 10 g scale, giving **3a** in excellent isolated yield (87%), with only small amounts of polybrominated byproducts as contaminants (<2%). The pure product was obtained through (unoptimised) crystallisation from toluene, giving **3a** in 80% overall yield, and undetectable ^1^H NMR impurities.

The application of the same protocol on *o*-xylene (**1**, Figure 3) cleanly gave dibromide **1a** in almost quantitative yield. Some issues emerged during the isolation step, because of the high lacrimatory activity of **1a** [53–54], while **3a** lacks the same effect.

Higher nucleophilic aromatic cores are affected by minor, yet inevitable, bromination on the ring. This is the case of dialkyl-substituted arenes having *o*,*p*-activated positions (*ortho* with respect to one alkyl substituent and *para* with respect to another alkyl substituent). For instance, bromination of *m-*xylene (**2**, Figure 3) resulted in lower selectivity for the benzyl α,α*’*-dibromide (**2a**) compared to **1a** or **3a**, due to the partial ring bromination [55]. The structure of mesitylene (**4**), featuring three *o,p*-activated positions, makes the selective halogenation of benzylic positions even more challenging. The conventional bromination with NBS in CCl_4_ yields no more than 30% of the desired 1,3,5-tris(bromomethyl)benzene (**4a**) [56], due to the concurrent ring bromination [57]. A modified method working in refluxing benzene and benzoyl peroxide initiator was claimed to provide a clean conversion to **4a** with very high yields [36,58]. However, in our hands, this procedure resulted in significant amounts of ring bromination too. Using the peroxide-bromide conditions developed for *p*-xylene, **4a** was obtained with a 42% yield. In this case, two chromatographic separations in sequence were necessary to remove most of the core brominated byproducts, that were still detected in small amounts through ^1^H NMR (see the image in the Supporting Information File 1).

Ring bromination of xylenes is commonly carried out using molecular bromine in the absence of light, along with the aforementioned catalytic promoters (FeBr_3_ or I_2_) [59–60]. Some reports of ring bromination by means of NBS or the peroxide-bromide method are also known, but mainly for highly activated arenes (phenols or anisoles, for instance) or for the synthesis of monobromides [49–50,61]. Continuing our investigation on the subject, the ring bromination of *p*-xylene (**3**, Figure 3) towards **3b** was considered. The starting conditions were taken from procedures established for monobromides [49,62], adapting the molar amount of the brominating agent (HBr·2H_2_O_2_) and including a small amount of CH_2_Cl_2_, according to the previous discussion. A catalytic amount of iodine (≈1 mol %) was also included. Under these conditions, a first assessment of the reaction time was performed (entries 1, 2, 3 of Table 2), resulting in the predominant ring monobromination, even after 48 h. Considering the continuous conversion of HBr into Br_2_ by means of the peroxide, we speculated on the decreasing acidity throughout the reaction progress. Since acidity was claimed to have a crucial role [63–64], we contemplated the addition of a small amount of sulphuric acid (Table 2, entry 4).

**Table 2:** *p*-Xylene bromination with in situ-generated Br_2_ in absence of light.^a^

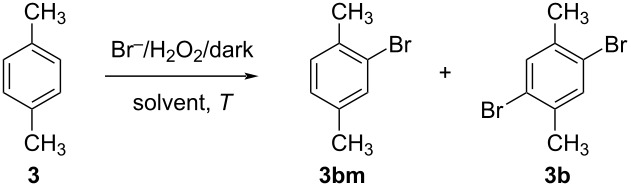						

entry	CH_2_Cl_2_ (mL)	*T* (°C)	*t* (h)	H_2_SO_4_ (mmol)	**3bm** (%)	**3b** (%)

1	1.5	rt	16	–	90	10
2	1.5	rt	24	–	75	25
3	1.5	rt	48	–	73	27
4	1.5	rt	48	0.5	50	50
5	1.5	rt	168	0.5	43	57
6	1.5	60	72	0.5	40	60
7	–	60	72	0.5	78	22
8^b^	1.5	60	48	11.5	14	86
9^b,c^	1.5	60	48	11.5	0	100

^a^Reaction conditions: **3** (531 mg, 0.62 mL, 5 mmol), I_2_ (15 mg, 0.07 mmol), HBr (48 wt % aqueous solution, 1.25 mL, 11 mmol), H_2_SO_4_ (98 wt %, see Table), H_2_O_2_ (35 wt % aqueous solution, 2.00 mL, 20.6 mmol) dropped in 2 h by a syringe pump, CH_2_Cl_2_ (see Table). Selectivity is reported as molar % obtained from ^1^H NMR spectra. ^b^NaBr (11 mmol) and H_2_SO_4_ (98 wt %, 11.5 mmol) instead of HBr (48 wt % aqueous solution). ^c^Changed reagent order: NaBr (11 mmol) dissolved in H_2_O_2_ (35 wt % aqueous solution, 2.00 mL, 20.6 mmol) dropped in 15 min to a mixture of **3** (5 mmol, solution in CH_2_Cl_2_ (1.5 mL) and H_2_SO_4_ (98 wt %, 11.5 mmol).

This adjustment led to a significant enhancement towards **3b**, resulting in a respectable 57 mol % selectivity, after prolonged reaction time (Table 2, entry 5). Furthermore, a slight improvement was observed at higher temperatures (Table 2, entry 6), while the removal of CH_2_Cl_2_, possibly limiting the temperature inside the flask, had a negative impact (entry 7). Nevertheless, yields and selectivity seemed to plateau around 60%.

Data available in the literature suggested a beneficial effect coming from the reduction of the water amount [62]. As all the existing water originates from the employed reagents like aqueous HBr and H_2_O_2_, the combination of NaBr and H_2_SO_4_ was explored as a potential source of anhydrous HBr. A notable increase in selectivity towards **3b** was suddenly observed (Table 2, entry 8). However, this method led to the undesired, uncontrolled generation of Br_2_ when neat H_2_SO_4_ was mixed with NaBr. This issue was addressed through an alternative reagent introduction scheme, where NaBr was dissolved in aqueous hydrogen peroxide and was gradually added to the reaction mixture, containing the remaining chemicals. The addition time of this aqueous reagent was also shortened to 15 minutes, to counteract the slow decomposition of H_2_O_2_ caused by NaBr. This modification ultimately resulted in nearly complete conversion of **3** into **3b** (Table 2, entry 9).

The method was successfully scaled up to 10 g of substrate, giving 1,4-dibromo-2,5-dimethylbenzene (**3b**) in quantitative isolated yield. The method was promptly extended to the other xylenes and to mesitylene, giving bromides **1b**, **2b**, and **4b** in excellent isolated yields.

An evaluation of the E factors involved in the here developed benzylic bromination towards **3a** (Figure S1 in Supporting Information File 1) and ring bromination towards **1b** (Figure S2) was done, based on the 10-gram scale procedures (see Supporting Information File 1). The obtained values, both inferior to 4.5 (i.e. less than 4.5 kg of wastes to get 1 kg of product), denotes a good sustainability. Moreover, more than 2 kg of wastes come from losses in the recycle of CH_2_Cl_2_. Therefore, these results can be easily improved by increasing the efficiency of solvent recovery, here set at 85%.

The aryl-cored bromides featuring both core and side chain halogens (**1c**, **3c**, and **4c** of Figure 3) are also of interest for the chemical community. The preparation of these polybromides can be ideally approached with two synthetic strategies: first-benzylic-then-ring or first-ring-then-benzylic halogenation. Of the two alternatives, the latter is more advantageous, due to three favourable features [65–66]: (i) Optimal control as each ring halogenation inhibits further ring halogenations, (ii) ring halogenation do not negatively affect benzylic bromination, and (iii) aryl bromides often display better solubility in organic solvents than benzyl bromides, reducing precipitation issues. Applying now the first-ring-then-benzylic halogenation strategy to *p*-xylene, dibromide **3b** was successfully converted into **3c**, by means of the method herein developed for the benzylic bromination. The process was then telescoped, resulting in a one-pot, two-step synthesis of **3c** from **3**, with improved operativity and yield (see experimental part). The same strategies were followed for the preparation of **1c** (from **1b** or from **1**), as well as for **4c** (from **4b** or from **4**). The preparation of the tetrabromo compound **2c** was instead not investigated, due to low SciFinder^®^ score (Figure 3).

The nucleophilicity of **4** provides useful support for its ring bromination towards **4b**, resulting in an excellent isolated yield of 89%. Moreover, the intermediacy of **4b** is advantageous for obtaining **4c**, thus avoiding the selectivity issues observed during the benzylic bromination of **4**. As a result, **4c** was obtained from **4** (in a one-pot, two-step process) with an 85% isolated yield. The particular cleanliness of the conversion of **4b** into **4c**, featuring negligible formation of gem dihalides, could arise from the useful steric hindrance given by the halogen bound to the core.

**Figure 4:**
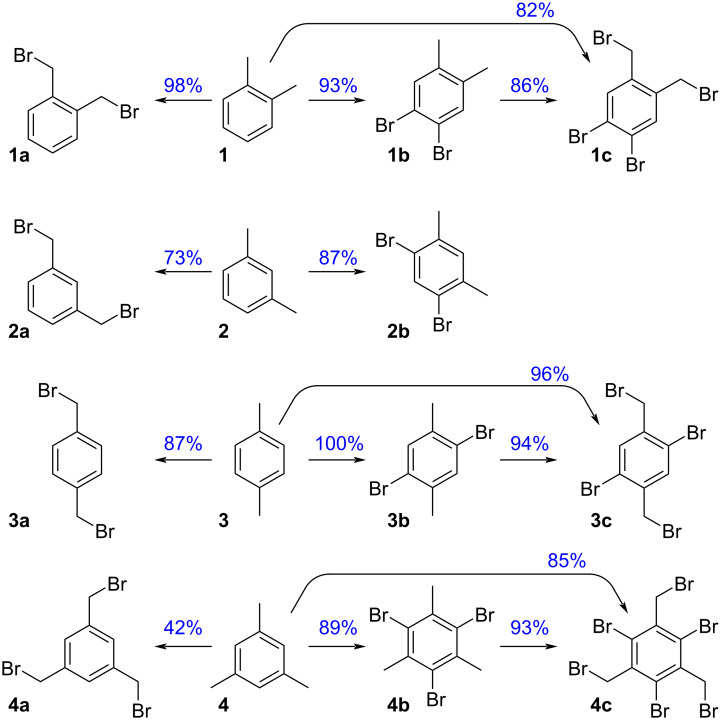
Yields obtained in the preparation of aryl-cored halides.

Figure 4 collects the results obtained in the preparation of aryl-cored halides of Figure 3, by means of the peroxide-bromide process. For products featuring bromine on both the aromatic core and the side chain, the “direct” one-pot two-stage and the “indirect” first-ring-then-benzylic halogenation methods are compared.

## Conclusion

In summary, the peroxide-bromide halogenation method, originally developed for the monobromination of benzenoid structures, has been extended to synthesize aryl-cored polybromides with high yield and high atom economy. This method, when used with light irradiation as halogen initiator, is capable of selectively convert xylene isomers and mesitylene into their corresponding benzyl bromides, regardless the presence of halogen atoms on the core. Moreover, under dark conditions, a modification of the same method allows the preparation of aryl polybromides through ring halogenation. The two variants, tested on a 10-gram scale, can be telescoped to achieve polybromo derivatives that feature both core and side chain substitution, in high yield. The application of this method enables the effective preparation of high-interest building blocks for polymer and materials chemists, offering improved sustainability compared to standard methodologies.

## Experimental

### General

Solvents and reagents were commercial grade and used as received. ^1^H NMR spectra were acquired with a Bruker Avance 400 spectrometer (Billerica, MA, USA). The lighting was achieved by means of a standard household white LED bulb (OSRAM 6.5 W, 2700 K, 806 lm). The H_2_O_2_ solution was dispensed by means of a syringe pump, mod. SyringeOne NE-300, from NewEra Instruments.

All the aryl-cored halides here prepared are known, and thoroughly characterized in [6] (compounds **1a**, **2a**, **3a**), [67] (compounds **1b**, **3b**, **4b**), [60] (compounds **2b**, **3b**, **3c**), [68] (compound **1c**), [56] (compound **4a**), and [69] (compound **4c**).

### General procedure for benzylic bromination

In a 15 mL Schlenk tube with screw cap, equipped with a magnetic stirring bar, the substrate (5 mmol, 1.0 equiv), solvent (either CH_2_Cl_2_ or chlorobenzene, 1.0–4.0 mL), H_2_O (1.5 mL), and HBr (48 wt % aqueous solution, *d* = 1.49 g/mL, 1.25 mL, 11 mmol, 2.2 equiv) were inserted. The mixture was kept under stirring at rt and irradiated with a LED lightbulb placed at 10 cm from the side of the reaction tube. Aqueous H_2_O_2_ (35 wt % solution, *d* = 1.13 g/mL, 2.00 mL, 23.3 mmol, 4.7 equiv) was added over 2 h using a syringe pump, through a small PTFE tube inserted through the side arm of the Schlenk tube. After the addition was complete, the mixture was left under stirring for 1 h and 30 min. Once the mixture was neutralised with solid NaHCO_3_, the product was extracted with CH_2_Cl_2_ (3 × 5 mL). The combined organic layers were concentrated to dryness to give the crude product.

### General procedure for ring bromination

In a 25 mL round-bottomed flask, equipped with a magnetic stirring bar, the substrate (12 mmol, 1.0 equiv), H_2_SO_4_ (96 wt %, 3.00 g, 30 mmol, 2.5 equiv), I_2_ (0.04 g, 0.16 mmol, 0.01 equiv), and CH_2_Cl_2_ (1.50 mL) were inserted. The flask, kept under stirring at room temperature (rt), was light-shielded with aluminium foil and a water-cooled condenser was installed on its top. A solution of NaBr (2.88 g, 28 mmol, 2.33 equiv) in H_2_O_2_ (35 wt % aqueous solution, 4.8 mL, 56 mmol, 4.67 equiv) was added to the mixture over 15 min with a syringe pump, through a PTFE tube inserted in the top of the condenser. The system was then refluxed for 48 h. Once cooled to rt, the reaction mixture was neutralised with solid NaHCO_3,_ and the product was extracted with CH_2_Cl_2_ (3 × 5 mL). The combined organic layers were concentrated to dryness to give the crude product.

### General procedure for one-pot first-ring-then-benzylic bromination

In a 25 mL round-bottomed flask, equipped with a magnetic stirring bar, the substrate (12 mmol, 1.0 equiv), H_2_SO_4_ (96 wt %, 3.00 g, 30 mmol, 2.5 equiv), I_2_ (0.04 g, 0.16 mmol, 0.01 equiv), and chlorobenzene (8.0 mL) were inserted at rt. The flask was shielded from light by means of an aluminium foil and a water-cooled condenser was installed on its top. Once the stirring was started, a solution of NaBr (2.88 g, 28 mmol, 2.33 equiv) in H_2_O_2_ (35 wt % aqueous solution, 4.8 mL, 56 mmol, 4.67 equiv) was added over 15 min through a PTFE tube inserted at the top of the condenser, by means of a syringe pump. The flask was then heated to 50 °C for 48 h, cooled to rt, and unwrapped.

Under stirring at rt, H_2_O (3.6 mL) and HBr (48 wt % aqueous solution, 3.00 mL, 26.5 mmol, 2.2 equiv) were inserted. The mixture was then irradiated with a LED lightbulb, placed at 10 cm from the side of the flask and aqueous H_2_O_2_ (35 wt % solution, 4.8 mL, 56 mmol, 4.7 equiv) was added over 2 h using a syringe pump, through a PTFE tube inserted at the top of the condenser. After the addition was complete, the mixture was left under stirring for 1 h and 30 min. The reaction mixture was then neutralised with solid NaHCO_3_ and the product was extracted with CH_2_Cl_2_ (3 × 10 mL). The combined organic layers were concentrated to dryness to give the crude product.

**1,2-Bis(bromomethyl)benzene** (**1a**): white solid, yield: 98%. ^1^H NMR (400 MHz, 298 K, CDCl_3_) δ 7.37 (m, 2H), 7.31 (m, 2H), 4.67 (s, 4H) ppm.

**1,2-Dibromo-4,5-dimethylbenzene** (**1b**): For the 10-gram scale procedure, see Figure S2 (Supporting Information File 1), brown solid, yield: 93%. Recrystallisation from hot petroleum ether gave the pure product. ^1^H NMR (400 MHz, 298 K, CDCl_3_) δ 7.37 (s, 2H), 2.19 (s, 6H) ppm.

**1,2-Dibromo-4,5-bis(bromomethyl)benzene** (**1c**): From **1b**: chlorobenzene (4.0 mL) was used as the solvent instead of CH_2_Cl_2_ (1 mL). Brown solid, yield: 86%. From **1** (two-step, one pot): Brown solid, yield: 82%. Recrystallisation from hot hexane gave the pure product. White solid, ^1^H NMR (400 MHz, 298 K, CDCl_3_) δ 7.62 (m, 2H), 4.53 (s, 4H) ppm.

**1,3-Bis(bromomethyl)benzene** (**2a**): Petroleum ether (1.5 mL) was used as the solvent instead of CH_2_Cl_2_. The reaction mixture was kept at 5 °C instead of rt and time was extended by 15% (2 h 20 min for H_2_O_2_ dropping and 2 h after the addition). The title product was obtained as pale-yellow solid, yield: 73%. Recrystallisation from hot petroleum ether gave the pure product. White solid, ^1^H NMR (400 MHz, 298 K, CDCl_3_) δ 7.42 (m, 1H), 7.33 (m, 1H), 7.32 (m, 2H), 4.48 (s, 4H) ppm.

**1,5-Dibromo-2,4-dimethylbenzene** (**2b**): Brown solid, yield: 87%. Recrystallisation from hot ethanol gave the pure product as pale-yellow solid. ^1^H NMR (400 MHz, 298 K, CDCl_3_) δ 7.68 (s, 1H), 7.10 (s, 1H), 2.31 (s, 6H) ppm.

**1,4-Bis(bromomethyl)benzene** (**3a**): For the 10-gram scale procedure, see Figure S1 (Supporting Information File 1), white-yellowish solid, yield: 87%. Recrystallisation from hot toluene gave the pure product as white solid. ^1^H NMR (400 MHz, 298 K, CDCl_3_) δ 7.37 (s, 4H), 4.48 (s, 4H) ppm.

**1,4-Dibromo-2,5-dimethylbenzene** (**3b**): pale orange solid, yield: quantitative. Recrystallisation from hot hexane gave the pure product as pale-yellow solid. ^1^H NMR (400 MHz, 298 K, CDCl_3_) δ 7.39 (s, 2H), 2.33 (s, 6H) ppm.

**1,4-Dibromo-2,5-bis(bromomethyl)benzene** (**3c**): From **3b**: chlorobenzene (4.0 mL) was used as the solvent instead of CH_2_Cl_2_ (1.0 mL). Pale-yellow solid, yield: 94%. From **3** (two-step, one pot): yellow solid, yield 96%. Recrystallisation from hot petroleum ether gave the pure product as white solid. ^1^H NMR (400 MHz, 298 K, CDCl_3_) δ 7.66 (s, 2H), 4.51 (s, 4H) ppm.

**1,3,5-Tris(bromomethyl)benzene** (**4a**): The general procedure was adapted considering the three benzylic positions. In a 15 mL Schlenk tube with screw cap, equipped with a magnetic stirring bar, substrate **4** (5 mmol, 1.0 equiv), CH_2_Cl_2_ (2.0 mL), H_2_O (2.0 mL), and HBr (48 wt % aqueous solution, *d* = 1.49 g/mL, 1.87 mL, 16.5 mmol, 3.3 equiv) were inserted. The mixture was kept under stirring at rt and irradiated with a LED lightbulb placed at 10 cm from the side of the reaction tube. Aqueous H_2_O_2_ (35 wt % solution, 3.0 mL, 23.3 mmol, 4.7 equiv) was added over 2 h using a syringe pump, through a small PTFE tube inserted through the side arm of the Schlenk. After the addition was complete, the mixture was left under stirring for 1 h and 30 min. After the workup, operated as described previously, a brown slurry was obtained. Yield: 42%. The crude product was purified via column chromatography on silica gel 60, eluting with petroleum ether. The pure product appears as a white solid. ^1^H NMR (400 MHz, 298 K, CDCl_3_) δ 7.35 (s, 3H), 4.45 (s, 6H) ppm.

**1,3,5-Tribromo-2,4,6-trimethylbenzene** (**4b**): The general procedure was adapted considering the three ring positions to be halogenated (3.5 equiv of NaBr were used instead of 2.33 equiv). The raw product appears as pale-yellow solid, yield: 89%. Recrystallisation from hot chloroform gave the pure product as white solid. ^1^H NMR (400 MHz, 298 K, CDCl_3_) δ 2.65 (s, 9H) ppm.

**1,3,5-Tribromo-2,4,6-tris(bromomethyl)benzene** (**4c**): The general procedure was adapted considering the three ring and the three benzylic positions. From **4b**: White solid, yield: 93%. From **4** (two-step, one pot): Pale yellow solid, yield 85%. ^1^H NMR (400 MHz, 298 K, CDCl_3_) δ 4.92 (s, 6H) ppm.

## Supporting Information

File 1 Addtional figures and tables and copies of spectra.

## Data Availability

All data that supports the findings of this study is available in the published article and/or the supporting information to this article.
