# Supplementary material for: Light on the sustainable preparation of aryl-cored dibromides
Source: Beilstein J Org Chem. 2024 May 14;20:1076–87. doi: 10.3762/bjoc.20.95 (PMC11106674; doi:10.3762/bjoc.20.95)

BEILSTEIN JOURNAL OF ORGANIC CHEMISTRY

Supporting Information

for

Light on the sustainable preparation of aryl-cored dibromides

Fabrizio Roncaglia, Alberto Ughetti, Nicola Porcelli, Biagio Anderlini, Andrea Severini and Luca Rigamonti

Beilstein J. Org. Chem. **2024,** *20,* 1076–1087. doi:10.3762/bjoc.20.95

Addtional figures and tables and copies of spectra

License and Terms: This is a supporting information file under the terms of the Creative Commons Attribution License (https://creativecommons.org/licenses/by/4.0). Please note that the reuse, redistribution and reproduction in particular requires that the author(s) and source are credited and that individual graphics may be subject to special legal provisions.
The license is subject to the *Beilstein Journal of Organic Chemistry* terms and conditions: (https://www.beilstein-journals.org/bjoc/terms)

Table S1 – Preparation of **3a** from **3**, using variable H_2O_2 amounts.

entry	H_2O_2 35 wt% (mL)	unconverted **3**	**3am** (%)	**3a** (%)	**3at** (%)
1	1.0	9	11	80	-
2	1.2	8	8	84	-
3	1.5	5	3	86	-
4	2.0	-	1	90	6
5	2.2	-	1	90	9

Reaction conditions: *p*-xylene (531 mg, 5 mmol), H_2O (2.0 mL), HBr (48 wt% aqueous solution, 1.24 mL, 11 mmol), Osram white LED lamp 6.5 W, H_2O_2 (35 wt% aqueous solution, dropped at 1.0 mL/h by a syringe pump), CH_2Cl_2 (0.5 mL).

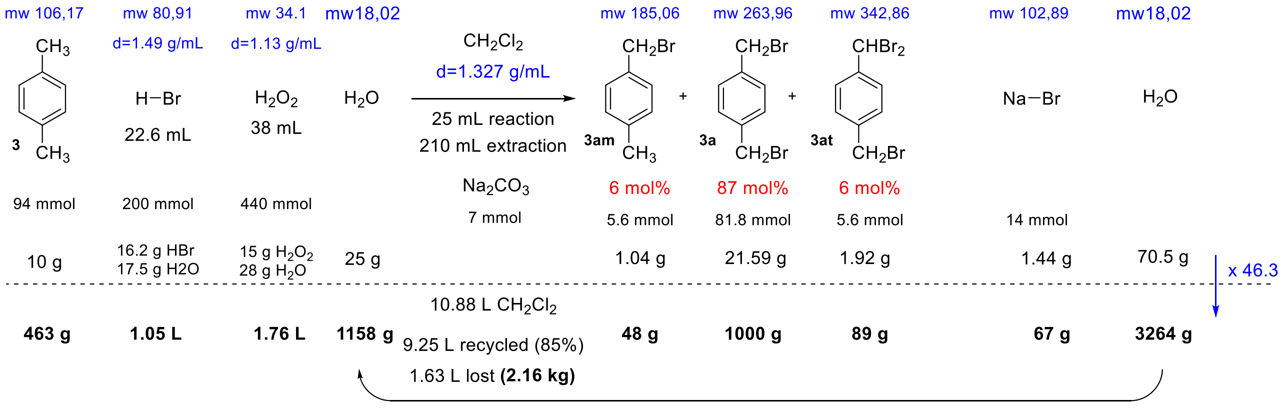

$$E = \frac{2.11\ \text{kg}\ H_2O + 0.14\ \text{kg by-products} + 0.07\ \text{kg NaBr} + 2.16\ \text{kg}\ CH_2Cl_2}{1\ \text{kg of}\ \mathbf{3a}} = 4.46$$

Figure S1. Evaluation of E factor for the conversion (benzylic bromination) of **3** into **3a**, based on the 10-gram scale procedure: In a 150 mL Schlenk tube with screw cap, equipped with a magnetic stirring bar, substrate (10 g, 94 mmol, 1.0 equiv), CH_2Cl_2 (25 mL), H_2O (25 mL) and HBr (48 wt % aqueous solution, *d* = 1.49 g/mL, 22.6 mL, 200 mmol, 2.13 equiv) were inserted. The mixture was kept under stirring at rt and irradiated with a LED lightbulb placed at 10 cm from the side of the reaction tube. Aqueous H_2O_2 (35 wt % solution, *d* = 1.13 g/mL, 38 mL, 440 mmol, 4.7 equiv) was added over 2 h using a syringe pump, through a small PTFE tube inserted through the side arm of the Schlenk. After the addition was over, the mixture was irradiated under stirring for 1 h and 30 min. Once the mixture was neutralized with solid Na_2CO_3 (0.74 g, 7 mmol) the product was extracted with CH_2Cl_2 (3 × 70 mL). The combined organic layers were concentrated to dryness to give the crude product as white-yellowish solid in 87% yield. The solvent recovery efficiency was set at 85 wt %.

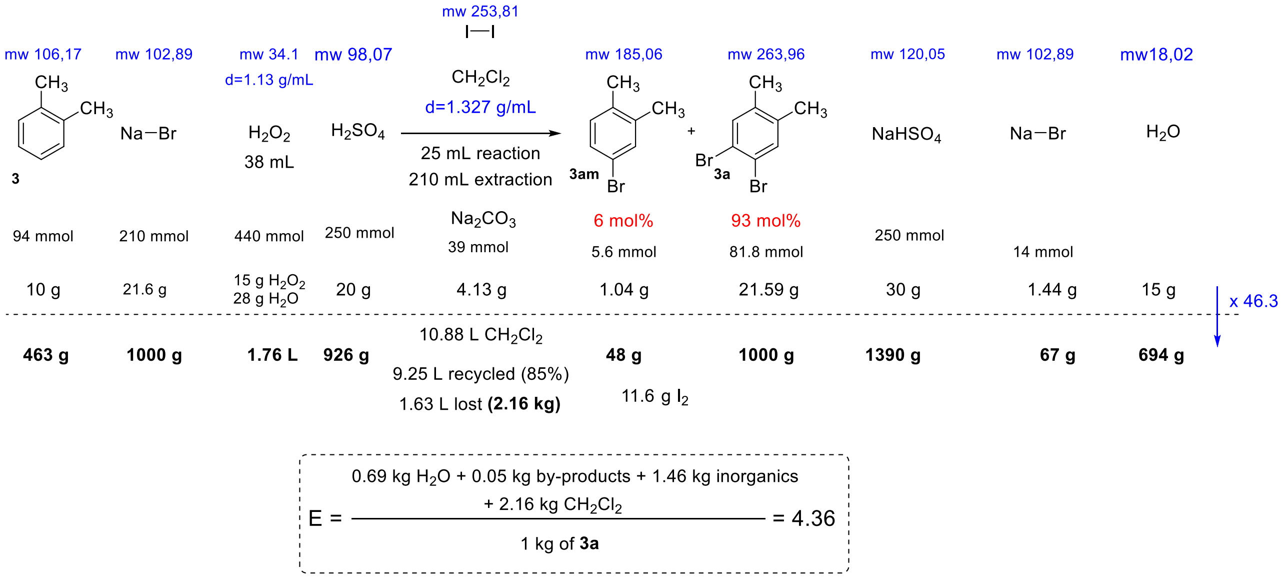

Figure S2. Evaluation of E factor for the conversion (ring bromination) of **1** into **1b**, based on the 10-gram scale procedure: In a 100 mL round-bottom flask, equipped with a magnetic stirring bar, substrate (10 g, 94 mmol, 1.0 equiv), H_2SO_4 (96 wt %, 20.0 g, 235 mmol, 2.4 equiv), I_2 (0.25 g, 1 mol %) and CH_2Cl_2 (25 mL) were inserted. The flask, kept under stirring at rt, was light-shielded with aluminium foil and a water-cooled condenser was installed on its top. A solution of NaBr (2.88 g, 28 mmol, 2.33 equiv) in H_2O_2 (35 wt % aqueous solution, 38 mL, 440 mmol, 4.7 equiv) was added to the mixture over 20 min with a syringe pump, through a PTFE tube inserted in the top of the condenser. The system was then refluxed for 48 h. Once cooled to RT, the reaction mixture was neutralized with solid Na_2CO_3 (4.13 g, 39 mmol), and the product was extracted with CH_2Cl_2 (3 × 70 mL). The combined organic layers were concentrated to dryness to give the crude product as brown solid in 93% yield. The solvent recovery efficiency was set at 85 wt %.

^{1}H NMR spectra (400 MHz, 298 K, $CDCl_3$)

All the synthesized compounds correspond to well-known structures. In the following, the ^{1}H NMR spectra of the extracted reaction mixture are reported. For compound **3a**, the spectrum for the crystallized product is reported. Compound **4a** from the raw reaction mixture was strongly contaminated by aryl bromides by-products (see main text). The reported spectrum is that obtained after the first chromatographic purification. A purer version is obtainable after a second purification, resulting in 42% yield (see main text).

1,2-Bis(bromomethyl)benzene (**1a**): ^{1}H NMR (400 MHz, 298 K, $CDCl_3$): δ 7.37 (m, 2H), 7.31 (m, 2H), 4.67 (s, 4H) ppm.

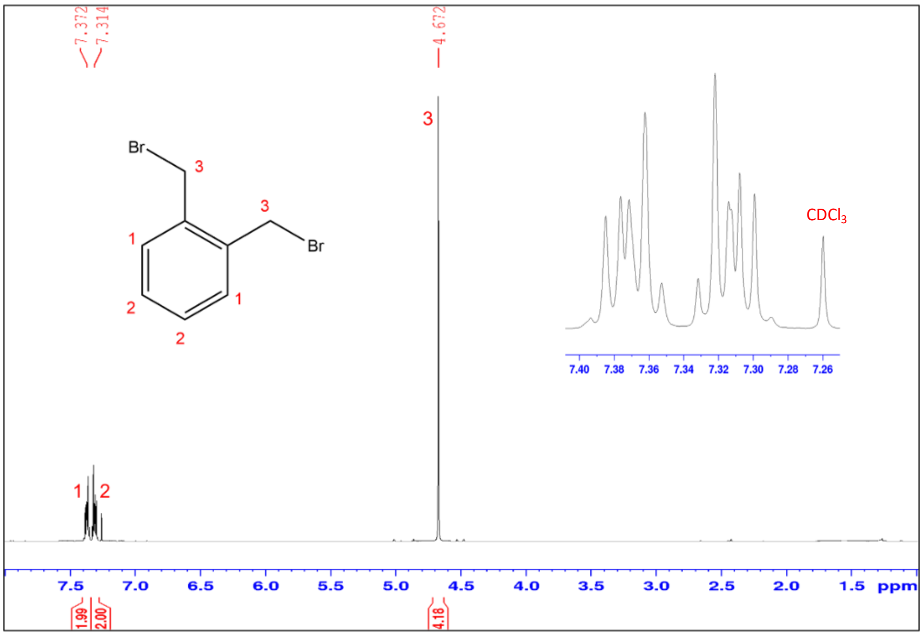

1,2-Dibromo-4,5-dimethylbenzene (**1b**): ^{1}H NMR (400 MHz, 298 K, $CDCl_3$) δ 7.37 (s, 2H), 2.19 (s, 6H) ppm.

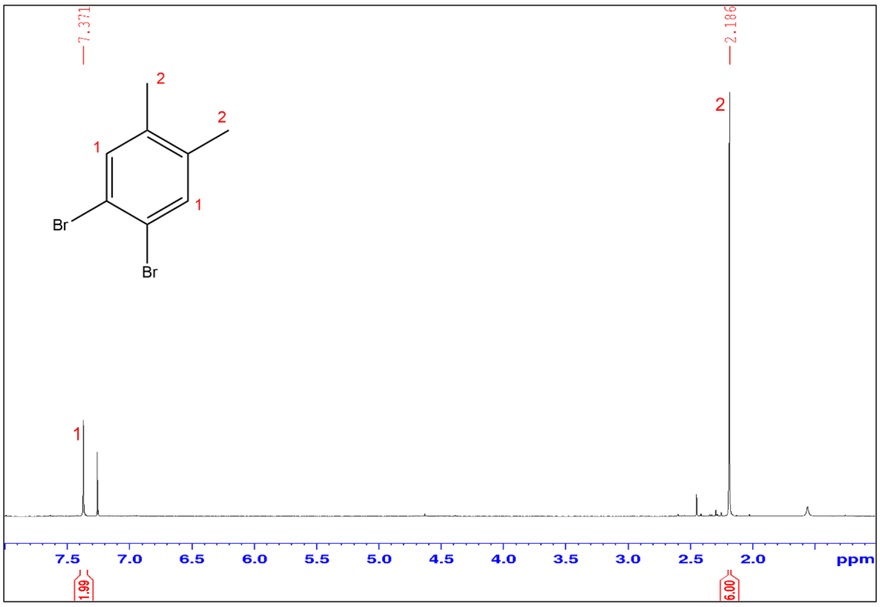

1,2-Dibromo-4,5-bis(bromomethyl)benzene (**1c**): ^{1}H NMR (400 MHz, 298 K, $CDCl_3$): δ 7.62 (m, 2H), 4.53 (s, 4H) ppm.

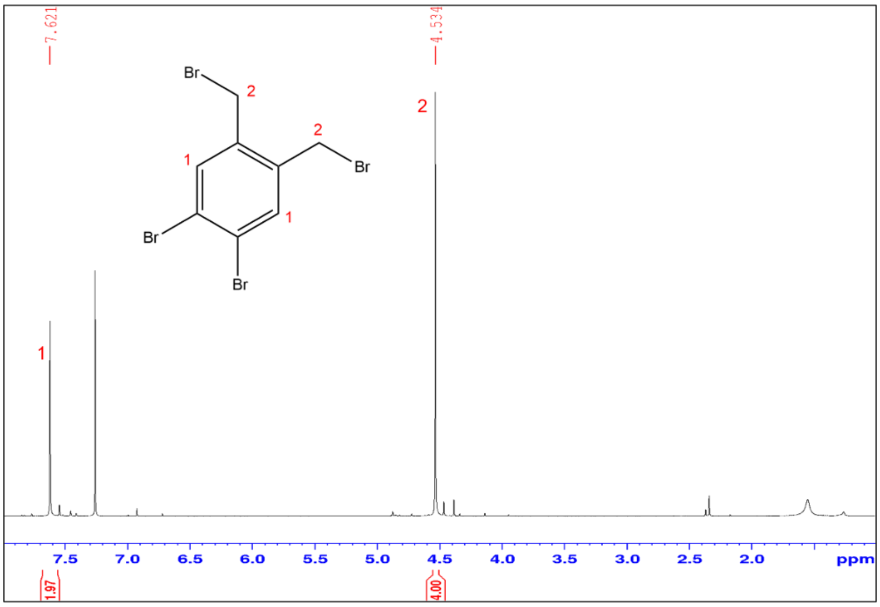

1,3-Bis(bromomethyl)benzene (**2a**): ^{1}H NMR (400 MHz, 298 K, $CDCl_3$): δ 7.42 (m, 1H), 7.33 (m, 1H), 7.32 (m, 2H), 4.48 (s, 4H) ppm.

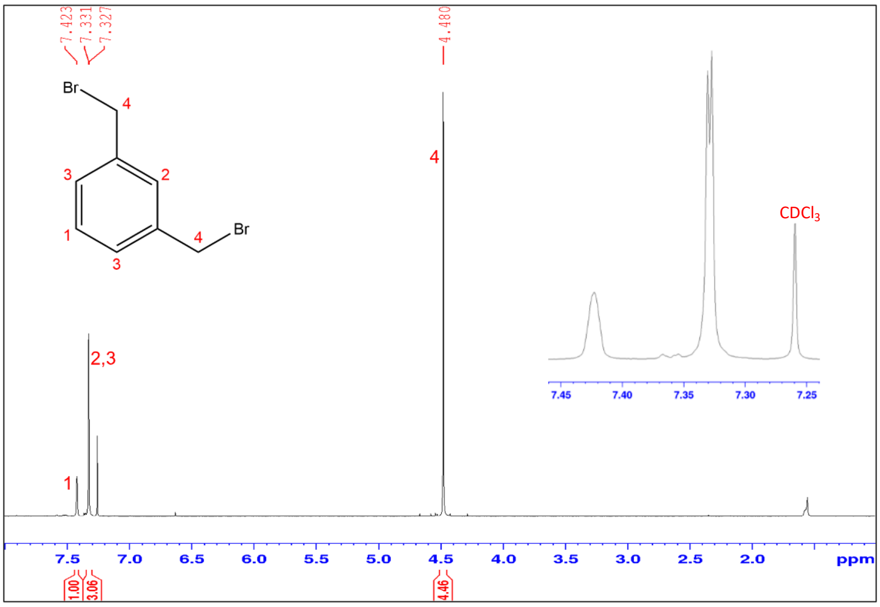

1,5-Dibromo-2,4-dimethylbenzene (**2b**): ^{1}H NMR (400 MHz, 298 K, $CDCl_3$): δ 7.68 (s, 1H), 7.10(s, 1H), 2.31 (s, 6H) ppm.

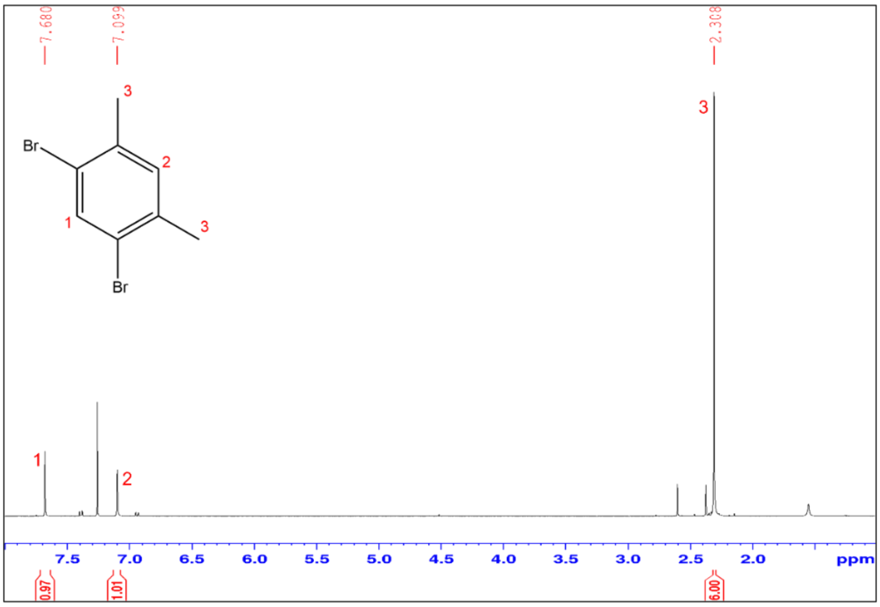

1,4-Bis(bromomethyl)benzene (**3a**) (crystallized from toluene): ^{1}H NMR (400 MHz, 298 K, $CDCl_3$): δ 7.37 (s, 4H), 4.48 (s, 4H) ppm.

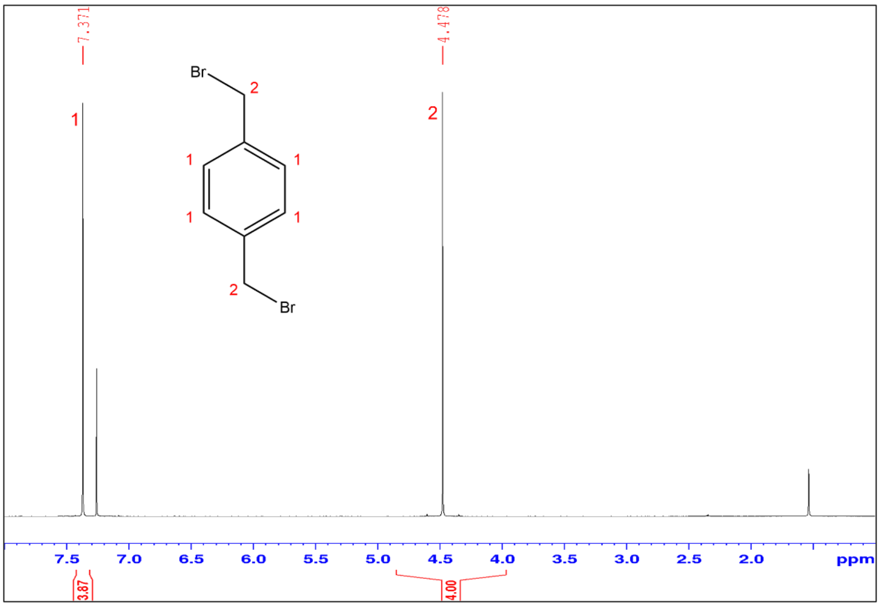

1,4-Dibromo-2,5-dimethylbenzene (**3b**): ^{1}H NMR (400 MHz, 298 K, $CDCl_3$): δ 7.39 (s, 2H), 2.33 (s, 6H) ppm.

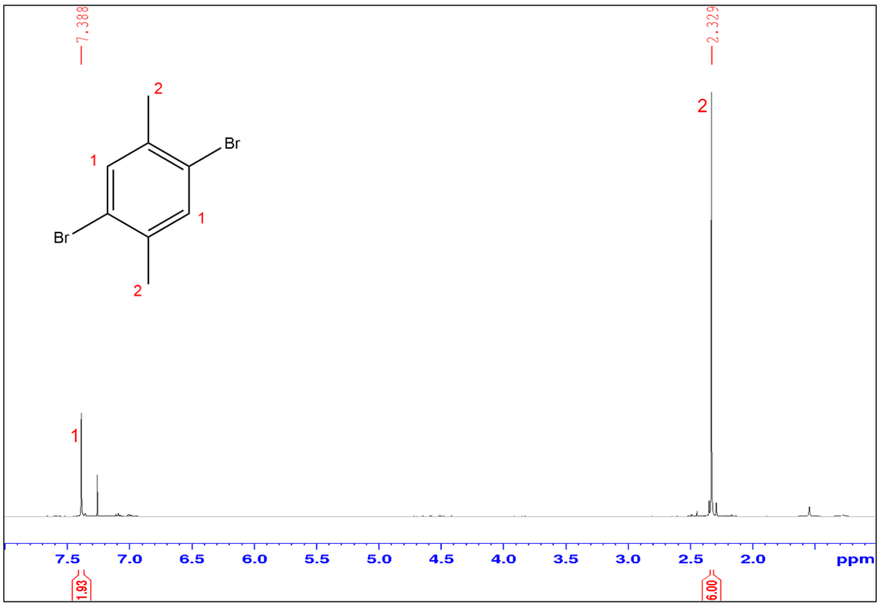

1,4-Dibromo-2,5-bis(bromomethyl)benzene (**3c**): ^{1}H NMR (400 MHz, 298 K, $CDCl_3$): δ 7.66 (s, 2H), 4.51 (s, 4H) ppm.

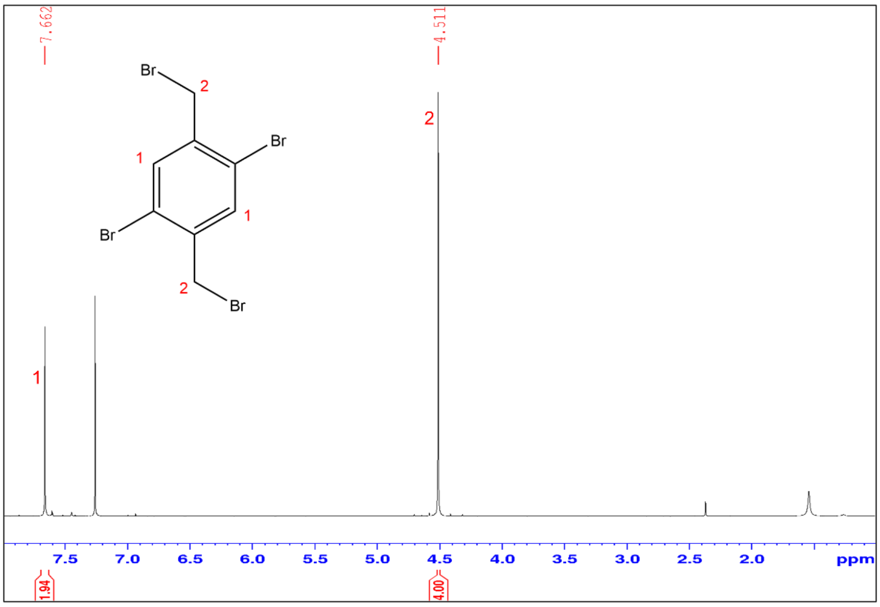

1,3,5-Tris(bromomethyl)benzene (**4a**) (after column chromatography): ^{1}H NMR (400 MHz, 298 K, $CDCl_3$): δ 7.35 (s, 3H), 4.45 (s, 6H) ppm.

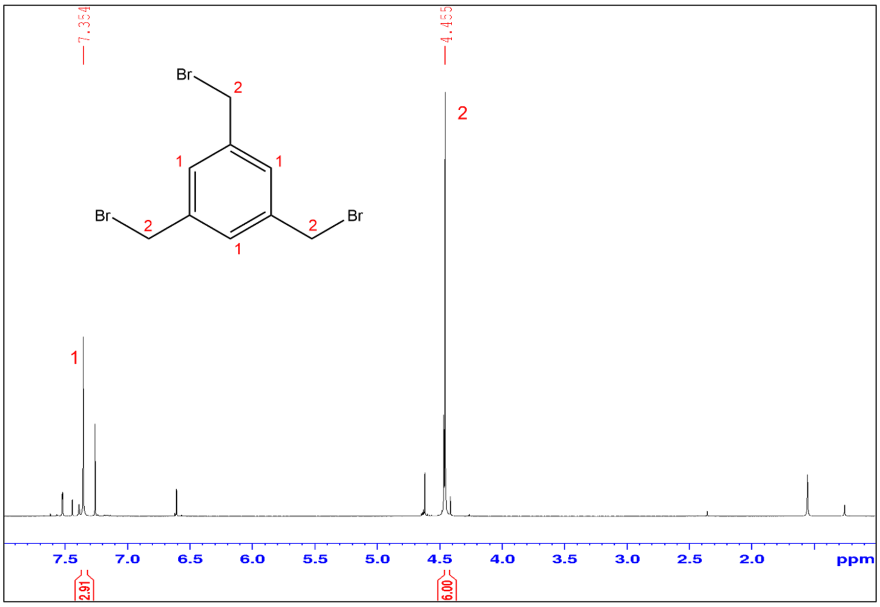

1,3,5-Tribromo-2,4,6-trimethylbenzene (**4b**): ^{1}H NMR (400 MHz, 298 K, $CDCl_3$): δ 2.65 (s, 9H) ppm.

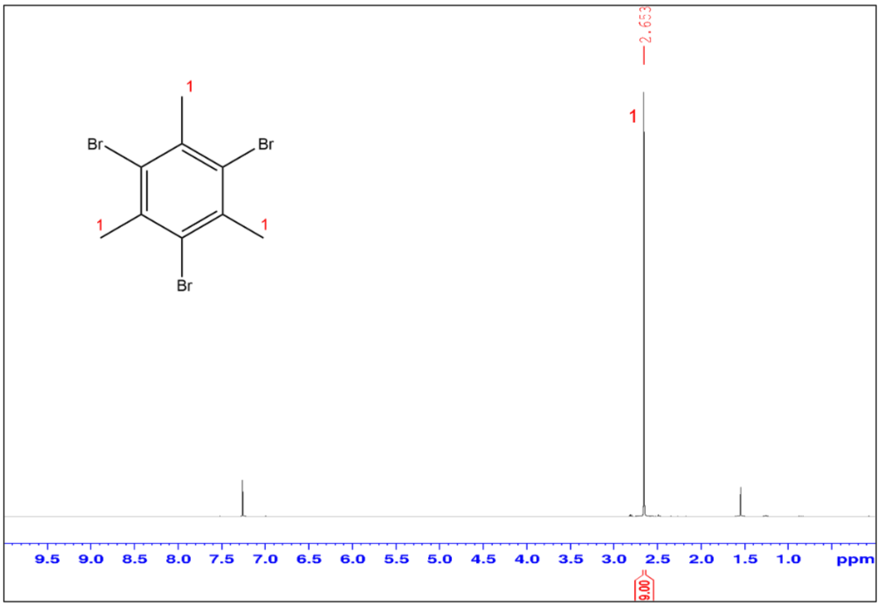

1,3,5-Tribromo-2,4,6-tris(bromomethyl)benzene (**4c**): ^{1}H NMR (400 MHz, 298 K, $CDCl_3$): δ 4.92 (s, 6H) ppm.

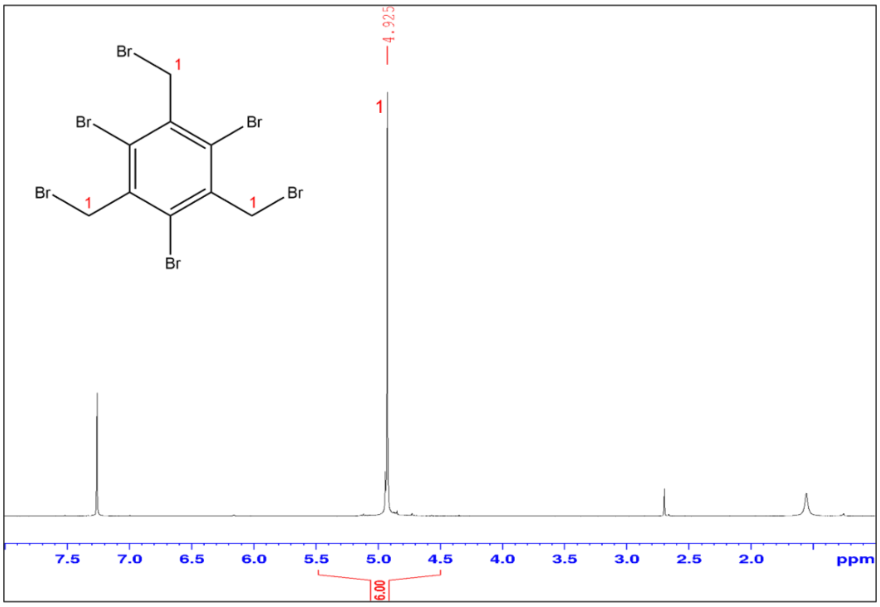

Supplement: File 1 — Addtional figures and tables and copies of spectra. [file Beilstein_J_Org_Chem-20-1076-s001.pdf]
